# Player Experience During the Junior to Senior Transition in Professional Football: A Longitudinal Case Study

**DOI:** 10.3389/fpsyg.2020.01672

**Published:** 2020-07-09

**Authors:** Scott C. Swainston, Mark R. Wilson, Martin I. Jones

**Affiliations:** Sport and Health Sciences, University of Exeter, Exeter, United Kingdom

**Keywords:** video diaries, longitudinal, talent development, elite sport, within career transition, soccer

## Abstract

The purpose of this study was to explore the evolving perspectives of young players experiences going through the junior to senior transition in professional football. A primary objective was to adopt novel methods – weekly video diaries – to allow participants to control and report their own narratives as the transition unfolded over 40 weeks. Semi structured interviews, held at four time points, allowed the lead researcher to probe further on themes that were developing. Six participants from the academy volunteered to take part, but only the three who earned professional contracts completed the study. The primary themes in the academy were the pressure experienced waiting for the contract decision, and then preparation for senior football and the first team environment once contracts were awarded. Adaptation to senior football included not only increased physical and mental demands but also those related to the different style of play, the pressure to win, and how these both impacted decision-making. The football club set up two pathways to support this adaptation, loan moves and time with the U23’s. In the following season, the move to the senior squad was characterized by a lack of opportunity to play for the first team, resulting in additional loan moves. These moves, and the associated perceived lack of support structures, led to the participants experiencing issues with their club identity, their motivation and their confidence. Internal (mindset) and external (social support) coping strategies were developed over the study’s duration. Concluding comments from participants were related to greater acceptance of the need to be patient, perhaps reflecting on the club’s reputation of giving young players a sound football education. These phases of the transition came with ups and downs for each participant illuminating key elements of the adaptation to senior competition, barriers to transition without early success, and social aspects of the transition. Enhanced detail to these key areas poses important questions for future research and applied practice.

## Introduction

Every year, hundreds of footballers across Europe are offered their first professional contract. However, very little research explores the unique and challenging experiences that players go through as they step up from the academy into the first team (Morris et al., 2016b). Stambulova et al. (2009) stated that this junior to senior transition is the most challenging step in a player’s career, and may take up to 5 years, as players orientate, adapt, and hopefully stabilize themselves within their senior teams (Stambulova et al., 2017). There is more to making a successful transition to the professional ranks than simply being a talented academy player (Gledhill et al., 2017), and indeed, youth team players appear to be unprepared for the challenges of the adaptation process during this transition, as outlined both by empirical research conclusions (e.g., Morris et al., 2016a) and by the opinion of top managers (e.g., Pep Guardiola in Ducker, 2017). The aim of the current study was, therefore, to examine the experience of players currently going through the junior-to-senior transition to develop a detailed understanding of their perceived challenges and opportunities.

A transition is defined as an event, or non-event, that leads individuals to change assumptions about themselves (Schlossberg, 1981). In transitioning from youth football to professional adult football, a player will face an increase in physical, technical, tactical, and psychosocial demands on the field of play (Haugaasen and Jordet, 2012). At the same time these changing assumptions will also pose additional challenges off the field of play (Wylleman and Lavallee, 2004). How an athlete copes with these demands and overcomes barriers, by accessing both internal and external resources, largely determines whether an athlete transition is successful (Stambulova, 2003).

A recent empirical model of the junior-to-senior transition by Stambulova and colleagues (Pehrson et al., 2017; Stambulova et al., 2017) explicitly considers the time course of the transition, which may take a number of years. Stambulova and colleagues identified four phases in their empirical model: preparation, orientation, adaptation, and stabilization. The preparation phase is defined as the preparation for a full-time move to the senior environment following initial experience with the senior squad. Following the full-time switch to the senior environment, the athlete begins the orientation phase where they learn about the new environment, how they fit in, and how they operate within the team or organizational structure. Once players learn the major features of senior sport they begin adapting to a bigger role in the squad in their performances and responsibility. Finally, players reach the stabilization phase as a regular member of the senior team (Pehrson et al., 2017; Stambulova et al., 2017).

The current study focuses on the first two of these phases, which is where the majority of the empirical studies in football have concentrated. Not only do these phases coincide with the specific event of the move from academy to senior football for most players, but also, this period marks some of the largest changes that are likely to occur in daily life and self-assumptions (Schlossberg, 1981). Morris et al. (2016a) interviewed footballers prior to and immediately after the start of their transition. They reported an almost immediate increase in motivation and confidence as well as a decreased sense of anxiety following the full time move to the first team. However, as the transition is not a single event but a process that occurs over time (Stambulova, 2003), it is critical to explore what happens after this initial positive feedback, as the subsequent challenges develop (e.g., Røynesdal et al., 2018).

Indeed, a recent position statement of the International Society of Sport Psychology explicitly calls for a better understanding of the changing demands across the transition (Stambulova et al., 2020). While we focus on these same two phases, our goal was to provide a more nuanced, deeper account of athlete experience as it unfolded before, during, and after this critical timepoint, through the use of weekly video diaries. This is especially important as Morris and colleagues have revealed that stakeholders in the transition believe there is a necessary period of adaptation that requires appropriate support and important personal characteristics in order to successfully navigate (Morris et al., 2016b; Drew et al., 2019).

Therefore, the purpose of the current study was to explore the evolving perspectives of young players experiences going through the junior to senior transition in professional football to develop a detailed understanding of the challenges during this process. As such, this study meets recent calls for more longitudinal research (Morris et al., 2016a, b) and the specific call from Drew et al. (2019) that research moves away from a reliance on one-off interviews. Additionally, it is hoped that the use of novel methodology for this research area – the thematic analysis of weekly video diaries – will provide a more detailed, nuanced and holistic understanding of this process. Such information is critical to further the research agenda in this field and help clubs support the development of future young footballers.

## Materials and Methods

### Methodological Rigor

At the outset of the proposed research we sought to develop methodological coherence as the primary source of rigor, through a reflexive, group process (Mayan, 2009). This process began with using the *armchair walkthrough* approach outlined by Mayan (2009). The longitudinal nature of this inquiry and the novel approach to data collection required a flexible framework, as in this instance there was no “one size fits all” structure as one might find with grounded theory or interpretative phenomenological analysis (Sparkes and Smith, 2009). The combination of longitudinal data collection techniques and semi-structured interviews provided broad and rich accounts of the player experience, demonstrating width of exploration. The inclusion of ample direct quotations represents the data in an honest fashion, in line with recommendations from qualitative description methodology (Sandelowski, 2010).

To further add rigor to our investigation we adopted criteria from Smith and Caddick (2012), specifically, coherence, transparency, resonance, and credibility. Credibility was established through the lead researcher being embedded in the environment to build established and trusting relationships between the researcher and participants. Video diaries allowed each participant to control which data they submitted to the lead researcher. Participants were able to pause, re-record, or delete any footage to ensure they were able to withdraw their data at the point of data collection rather than requesting to do so later in the study. The interviews then allowed the researcher to probe for deeper insights and feedback on interpretations. Transparency in the procedure and methods written below, as well as the theoretical positions of the research allow the reader to interpret the findings. While “external validity” is not sought in qualitative research our aim was to provide accounts that resonate with the reader and encourage comparisons to other contexts through naturalistic generalizability (Smith, 2018).

### Qualitative Description

We form our work from a naturalistic approach, attempting to study the phenomena in its natural context (Lincoln and Guba, 1985; Sandelowski, 2000, 2010). In line with our methodology, qualitative description, we attempted to allow the phenomena to emerge as if it were not under study (Sandelowski, 2000). The lead researcher (first author) was completing a Ph.D. which was partially funded by the football club. This role included spending several days a week at the football club and providing psychological support services to academy players. The lead researcher therefore had developed a working relationship with all of the academy players before the study started.

Our ontological perspective is based in relativism, in which we assume multiple realities and multiple truths to the phenomena in question (Denzin and Lincoln, 2011; Bradshaw et al., 2017). These multiple realities and truths are evident in the literal description of the thematic analysis by participant, showing the meaning each participant attributed to the transition (Sandelowski, 2010; Bradshaw et al., 2017). The epistemological assumption of constructionism is that knowledge is developed through subjective experience and socially constructed (Creswell and Poth, 2013). The collection of data in the everyday environment of the participants and lead researcher allowed for this subjective experience to be understood and described.

Qualitative description seeks to understand complex experience and processes through the use of multiple techniques of data collection. Participants should be purposively sampled to describe their experience while “in the midst” of the experience (Sullivan-Bolyai et al., 2005). The end goal is then to present the data in the purest, simplest form, with limited interpretation and a focus on the experiences the participants described (Sandelowski, 2000; Bradshaw et al., 2017). This is opposed to a hermeneutic phenomenological approach where interpretive meaning is given to experience (Neergaard et al., 2009). The simplistic, yet rich nature of the description should aid the data being used directly in an applied setting (Sandelowski, 2000, 2010; Sullivan-Bolyai et al., 2005). For these reasons, qualitative description was chosen as the method by which we sought to answer our research question: What are the evolving perspectives of young players experiences going through the junior-to-senior transition in professional football?

### Participants and Sampling

A total population sample of players in their second year of their 2-year apprenticeship within an English Football League club was used. Included participants were required to have been a part of the club’s academy for at least 3 years and have participated in at least one first team training session (i.e., the preparation phase of Stambulova et al., 2017 model). It is important to note that during the planning of this study it was unknown which of the participants would earn a contract and remain in the study. Traditionally, contracts are awarded at the end of the scholarship period in April, and data collection was planned accordingly. In total, six participants aged 17–18 years old (*M* = 17.85, *SD* = 0.38) agreed to participate in this study (see Table 1) from a total of nine who met the inclusion criteria. Participants had been in the academy between 4 and 10 years with an average length of (*M* = 8.5, *SD* = 1.9). At the halfway point of this study, the players’ 2-year apprenticeship ended. At this point three participants were released from the club while three continued with their first professional contract. The players who were released no longer continued their participation in the study.

**TABLE 1:** Participants’ (not their real names) demographic information.

**Name**	**Age**	**Time in academy**	**Contract status**
Oliver	18	4 years	Pro
David	18	10 years	Released
John	18	10 years	Released
Charlie	17	10 years	Pro
Thomas	18	8 years	Pro
George	18	8 years	Released

### Data Collection and Analysis

To better understand the participants’ evolving experiences, a longitudinal process was developed using both video diaries and semi-structured interviews. While multiple interview formats have value (e.g., Morris et al., 2016a) they result in timepoints of understanding rather than the evolving experience (Grossoehme and Lipstein, 2016). With this in mind we chose to use weekly video diaries to gain deeper insight into the experience of the participants throughout the ongoing transition. Rich et al. (2000) cited the effectiveness of video diaries in younger populations, based in part on their use in pop culture television (e.g., Big Brother). Further, familiarity with the technology will likely lead to young populations being more confident in sharing their personal stories (Rich et al., 2000).

**FIGURE 1:**
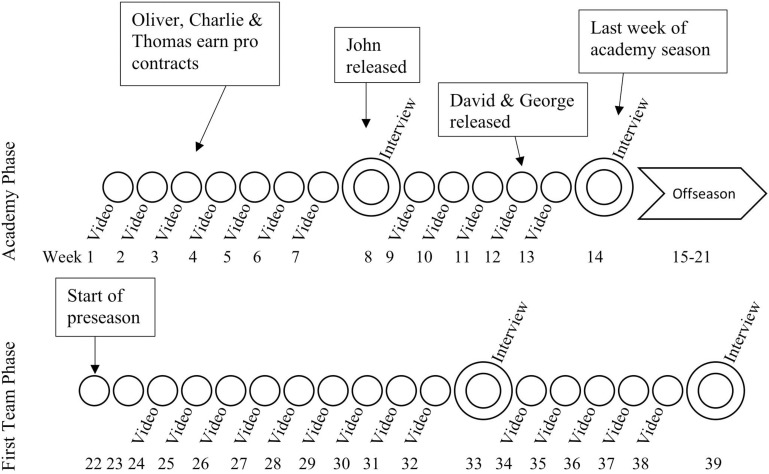
A timeline of the study protocol, outlining the key decision points and data collection methods.

Following ethical approval from the researchers’ institutional ethics committee, the lead researcher held a meeting to inform the eligible participants about the project and what would be required to participate. They were ensured that participation or non-participation would not affect their standing within the football club and all non-anonymized data would only be seen by the lead author and his supervisors (co-authors) and could not be accessed by coaches or other “gatekeepers” within the club. The data were collected on a weekly basis over 40 weeks. Weekly video diaries were supplemented by semi structured interviews (collected by the first author) roughly at the halfway point and at the conclusion of each phase of the study (see Figure 1 for a pictorial representation of the data collection timepoints over this period).

During the first week of data collection the lead researcher discussed with each participant the process of the video diaries and answered any questions. A well-known popular culture television show (Rich et al., 2000) was used as an example of a “video confessional” to guide participants in how to talk about their current experiences both on and off the pitch for roughly 5 min. An individual written guide was provided each week to prompt participants, but instructions on the guide indicated that they were not required to answer these questions and could discuss anything meaningful to them. Prompts were created in response to the data participants provided in previous video diaries. Each guide was different for every participant and changed weekly. Participants completed the video diaries during breaks in their day as full-time scholars at an English Football League club or at home during their free time. Video diaries were collected using the participants’ own mobile phone with printed questions to guide their diary reflections.

Following collection of the weekly video diary, the lead researcher transcribed and analyzed the transcript, following the first two steps of Braun and Clarke (2006) thematic analysis procedure. The collection and analysis of data followed an iterative approach where analysis preceded the collection of additional data. This iterative approach to data collection and analysis allowed for topics that were addressed in each week’s video diaries to be readdressed or clarified, and for the guides to build upon one another, adding depth to the data being collected. This process was repeated up to week seven, when codes were sorted, and interview guides were developed to discuss potential themes and each individual’s experience. The interviews allowed for potential themes that developed in the video diaries to be discussed in more detail. Interviews also enabled the lead researcher to probe using follow-up questions to gain further insight. The interview process also aided in the analysis of the data by allowing the lead researcher to better understand the position, opinion, and experience of each participant (Morse, 2020). The interview guides developed were highly personal, but also included general themes that had begun to develop across the participants.

This same iterative collection and analysis cycle was then repeated for the second half of the academy phase for all six participants and throughout the first team phase for the remaining three participants (see Table 2). Due to the differences in experience within the two phases of data collection they were separated in the final analysis. Codes for each phase were sorted into potential themes and reviewed against the coding by the lead researcher. The potential themes were discussed with the research team to ensure they accurately reflected the coding and the data. Using a thematic mapping technique (Braun and Clarke, 2006) higher and lower themes were developed for each phase. Once the higher and lower order themes were confirmed by the research team the report was generated with consideration given to the best way to represent the data.

**TABLE 2:** The number and mean duration of video diaries and interviews for each participant.

**Name**	**Oliver**	**Thomas**	**Charlie**	**David**	**George**	**John**
No. of video diaries	26	25	24	11	10	11
Average length	05:03	04:27	04:16	04:28	04:15	04:31
No. of interviews	4	4	4	2	2	2
Average length	32:05	28:12	26:41	38:04	25:50	22:52

### Data Representation

Results should be reported in a way that best fits the data (Sandelowski, 2000; Sullivan-Bolyai et al., 2005), whether that be by relevance or chronologically (Neergaard et al., 2009). With this in mind our data is presented chronologically by theme across the two different phases. We provide a straight description of the data giving voice to the participants’ experience with limited interruption (Sullivan-Bolyai et al., 2005). The presentation by theme allows similarities and differences to be shown between the participants giving the reader a full picture of the evolving transition.

## Results

The participants’ experiences were explored over two phases, while they were members of the academy (preparation; Stambulova et al., 2017) and following the full time move to the first team (orientation and adaptation; Stambulova et al., 2017). Themes were developed for each of these phases marked by the experience of those transitioning (see Table 3). In week three of the study Oliver, Charlie, and Thomas received early professional contracts. John was released in week eight, while David and George, were released at the end of the season (Figure 1). As a result, only the experience of the three players who made the transition are considered in this article^1^.

**TABLE 3:** Higher and lower order themes by phase.

**Phase**	**Higher order themes**	**Lower order themes**
Academy phase	Pressure of contract decision	Stress
		Motivation
	Adaptation to men’s football	Playing style
		Physicality
		Decision making
	Preparation for first team	Learning the demands
		Social adaptation
		Individual preparation
First team phase	First team environment	Social dynamic
		Organizational support
		First team demands
		Belonging
	Opportunity	Training experiences
		Loan opportunities
	Coping	Internal: Psychology/mindset
		External: Support network

### Academy Phase

#### Pressure

Pressure to earn a contract was inherent in the academy environment. Prior to receiving his early contract decision, Oliver discussed the stress he was experiencing, “before I got mine it was something I struggled with. The week I got told I had the meeting I couldn’t sleep for the whole week.” Charlie expressed a similar view, “I was worrying. It was affecting my performance. I was worrying about things I didn’t need to.” Pressure in general was also seen in a positive light by Oliver, “that pressure, as horrible as it feels, is good because it makes you want to improve.” This was evidenced in a key event in Thomas’s journey, “They said at the moment I’m not going to get (a contract) and something switched in my head mentally. I thought I have to do something now, change this because I didn’t have much time left.” He felt this helped him in establishing better habits, “I worked harder. I have done things more professionally, and that’s shown on the pitch with more consistency in games.” Oliver believed coping with the pressure was an important characteristic to demonstrate to coaches, “if you can get through that, then it shows that you will succeed.” Charlie discussed this in relation to his mindset, “you just have to do your best and they can’t expect more of you than to do your best.”

The early contracts eliminated this worry, allowing each to look forward to the next phase of their career. Oliver addressed this saying, “not that I can relax now, but I’m less stressed.” Specifically, when talking about the pressure on the pitch he commented, “it’s taken the pressure off me. It’s different, I know now that if I do something bad it won’t affect me too much. I can try more things and take more risks.” Although receiving his first professional contract provided a sense of security over his future, Charlie remained focused on his mindset, “It never stops, never stop working. Just because I have been told early and we have a few months left in the season, doesn’t mean I’m going to not play my best or relax.” This preparation period was the next focus for Oliver and Thomas. Oliver described, “I have already earnt it now, but what can I do when I start my pro? What can I do to push so when I come back, I’m fit and an even better player than I was before?” and Thomas stated, “obviously I have weaknesses that I still need to improve, that’s what I will be working on to be ready for next season.”

#### Adaptation to Senior Football

##### Adaptation via loan

Both Oliver and Thomas were sent on loan during the academy phase to begin their adaptation to men’s football. Both felt that this was an important development opportunity:

If I want to progress to a higher level of football, I need to do other parts of the game, the dirty parts. Where I am going to get kicked, I am going to get taken out, it’s not going to be fair, so I need to get used to it. (Oliver).

I think going against men will really test me mentally and physically. I feel that’s what I need right now to show the first team manager that I am capable of getting into his team. (Thomas).

Both were met with the realities of lower league football and the drastic differences when compared to academy football; “it’s completely different playing men’s football, their demands are different. It’s actually harder than I thought it would be.” (Oliver).

It’s “get it to the center half and boot it long,” which isn’t really my game. That’s proper football, that’s (lower) league football, that’s what I have to get used to so that’s why I think it’s a good level for me to get experience and grow as a player. (Thomas).

According to Thomas these differences required adaptation, “I think just learning about the ins and outs of football that you don’t get at the academy. It’s proper men’s football, it’s not really what I have been doing for the last 10 years.”

As the loan continued, Oliver and Thomas’s experiences separated as Oliver received regular playing time and Thomas did not. For Oliver, physical demands received less attention as his loan progressed. Instead tactical differences in senior football, that aren’t often seen in the academy, came to the fore.

We are used to playing football where it’s simpler into my feet or to the striker to play to me. Not too many times have I had to play off people where it’s flicks or play me in behind on the flick.

These differences required him to alter his decision making.

I think I am meeting the demands physically. It is more decision making, it’s more I have to hook a ball on or play it in behind and run after it. Which in 18’s football you have to do that, but you want to look after the ball a bit more.

He also added, “You have to learn to think your way around things.”

Another major difference for Oliver was the importance placed on winning matches: “Playing on loan, sometimes the best option is just to kick the ball up the pitch and leave it in the corner. That’s frustrating because I want to do something with it.” “You can be more spontaneous in 18’s football.” When summarizing his loan experience at the end of the season, Oliver reflected that it provided unique opportunities not present in academy football.

It’s hard to sometimes know what you want to do in those instinctive moments. Sometimes you don’t do the right thing but going on loan gives you experience. You get experience and confidence; you get confidence from that experience. I think the loan is really helping my transition.

Thomas’ lack of playing time on loan heavily influenced most of his experience during this phase, with less reflection on specific learning opportunities.

It’s probably just more frustrating, because when I was playing well for (the academy), I was playing week in and week out scoring goals. I was coming in happier and more confident, whereas now it’s a bit dull and I don’t want it to fizzle out.

However, in the final weeks of his loan he finally received an extended opportunity to play, helping his confidence prior to the offseason.

When you are looking at it on the sidelines, you think, look at these tackles and aerial battles, can I do this? It’s always going to be in the air and it’s not going to get on the floor. Then when I got out there, I proved to myself that I can handle that.

##### Adaptation via home club

Charlie was the only participant not to go on loan, resulting in a different adaptation pathway. The necessity to adapt to senior football was something he was aware of, “I think I have developed my game to suit (men’s) football more than it was last year. Last year it was to suit U18’s football. I feel now I have progressed my game to suit men’s football.” His only experience of men’s football was with the U23 team, yet this yielded at least one similar experience to Oliver’s loan.

It’s a different kind of football. U18’s matches you are passing the ball more. Saturday (with the U23’s) our game plan was two big strikers up front, one that was good at heading the ball and one that was quick. We were trying to play it up to one of them and flick it on for the other to run on to.

Charlie also mentioned conversations with the U23’s coach, also a first team coach, as helpful, “He speaks to us about what he wants in that game. What he expects from the formation, tactics, out of possession, and in possession. I think he sets you up like he would speak to a first team player.” “If (he) talks about the way we need to adapt our game to become senior football players, I think that’s a good thing for us.”

Another key area for Charlie was identifying differences between the U18’s and first team, “what they find is a good thing to do and what they find is something that needs to be better is going to be different than what you find at U18’s football.” Charlie elaborated, “you can quicken up your adaptation to the first team by watching their games back or spending more time with the first team if that’s possible.” As part of this learning process Charlie identified a senior player with a similar style of play to watch and learn from, “when we come off training we always see them doing bits of training, I always try to look out for what he does,” and when he attended first team matches, “I always try to see what he is doing. Trying to pick up ideas. He is very good at what he does, so if he does the things that are right then I can pick up on it.” Due to a period they were both injured at the same time Charlie had the opportunity to speak with him, “I was talking to him about what he finds to be the most important parts of his game to get into the team and what the manager wants.” He added,

I don’t see why players like us who are trying to learn from players like that can’t go up and speak to them. Ask them what they think. Someone like (senior player) who is like 35 and has 20 years’ experience in the game, I don’t see why you can’t go up to him and pick his brain a little bit.

#### Preparation for the First Team

##### Learning the demands

All three players spent limited time with the first team, trained once a week with the U23 group, and were included in several U23 matches. Through these experiences they developed perceptions on entering the first team environment and how they would need to prepare. Thomas suggested, “it’s just about getting experience really and making me feel more comfortable so when I step in it won’t be such a big surprise to me.” Charlie’s perspective was more specific, “I think everyone needs to get used to the way the first team do things. It’s different to ours (U18’s).” For Thomas, the experiences with the U23’s were beneficial in this regard, “I think this helps me in my transition by being around the pro’s. Seeing what they do in the changing room, and what it’s like to be in the first team changing room at the stadium.”

Preparing for an increased competitiveness was important to Oliver, “the toughness of it, the sort of drive that they all have. Every man for themselves kind of thing, even though you are a team. It’s the sense that you want to make a career for yourself.” “You have to be selfish in the fact that you can’t give it to someone else. Otherwise, that might be your only chance.” Oliver felt that entering this intense environment required a specific mindset. “I think the challenges of transition to the first team is not seeing myself as a lower player, like as a young one. That I see myself as a target to get into the team.” When asked more specifically about this he said, “I can’t see myself as a first-year pro, I have to see myself as a pro. My job is to try to play and play the best that I can and that hopefully will transition into one day playing with the first team.”

##### Social adaptation

Oliver also outlined some of the social dynamic concerns associated with entering this competitive environment. “I think you find it hard for those reasons and you are a young lad trying to take their place in the team. They won’t want that.” During the first week of the academy phase, Oliver explained his experience training up with the first team.

Socially, it’s different because you don’t have anyone around you that you can confide in. You are on your own for a bit and you have to make a friendship or make a bit of chemistry with one of the lads.

Thomas also believed the social aspects of the transition were important, “we are around the pros more often now and training with them more, getting to know them,” adding, “If it’s the first time seeing them you might be more nervous and just sit back, relax, and watch the session go by. If you are confident around them then you are going to show that on the pitch.” However, despite him saying it was important to get to know them, he added, “I don’t necessarily speak to anyone in the first team. Maybe I will more next year.”

Oliver reflected on his specific view of coping socially, which was to just improve as a footballer.

By being myself to be honest. I am not going to try to be someone I’m not. They will either get used to me or not like me, either way it doesn’t really matter as long as my football improves. That’s all I really care about.

##### Individual preparation

While social challenges were perceived to await, so too were challenges in gaining organizational emotional support. Thomas stated, “I think over there (first team), it’s harder to open up and speak to people, whereas here (academy) there is always someone to speak to. You can speak about your worries and they can help you.” However, despite changing support structures within the club, he felt he had good support from both his “natural” (“my mum is really supportive, even though I don’t see her much. She is always texting me asking how I’m getting on”) and host family (“they really look after me, I can tell them anything. They give me advice, listen, and they can recognize when I’m down”). Thomas did get informational support from the U18’s manager through the first team manager, “he has been feeding back to our coach what his thoughts are on games and in training. Which is good to know what he is thinking, so when we go over there isn’t not too much of a shock.”

Charlie had a proactive view on finding out important tactical information.

I can see in the next few months what the manager wants from (his positions). I think I can see what he wants from the player, what kind of tactics he wants them to do, parts of the game that they need to be good at. I can look at them and improve my game on that, and then hopefully get myself into the first team on that.

He came back to this point in later weeks discussing in more detail, “if you get to know him and what he likes, what he doesn’t like, what he finds would make a good or bad player then you know what you are getting really.”

Finally, Thomas and Oliver believed their experiences and focus on mental skills would benefit their move to the first team environment. Oliver discussed his view on psychology, “I think when you can get it right, get your head right it’s often the difference in consistency and how you play, how you improve. I think it’s helped me quite a bit.” Overall, he felt during the final months in the academy that he had developed a mindset that would aid in his transition.

Going out of your comfort zone makes you better. I’ve gotten in that mindset while being a scholar. It’s easy to just go with an easy group, whereas stepping out will be what I have to do at the start when I train with the first team. I am going to be out of my comfort zone, so those are the times that will make me ready.

Thomas recognized that moving away from home had helped in developing his character, “I have matured and grown into the lad that I am now because moving away from home is a big thing.” He also referenced how previous challenges helped him be more resilient when describing his younger years in the academy.

When you are down in the middle of the pack, they aren’t that bothered. I think going through that is quite hard. I was strong enough mentally. I have suffered not playing games and being on the bench all the time. There are people who have played their whole career and then something bad happens to them and they don’t know how to react to it. Whereas, it hasn’t always been positive with me and I’ve been strong enough to deal with that and react to it.

### First Team Phase

#### First Team Environment

##### First team demands

All three ended the academy phase having discussed their perceptions of what life might be like from their limited experience in the first team. Moving full-time to the first team environment came with experiences both confirming and refuting previous perceptions. Oliver ended the pre transition phase on a high and was met with a new reality at the start of the season.

It’s frustrating as a first-year pro. You are right down the bottom of the list of everything, so the chance you want or the chance you need, you might be pushed aside. Even if you do take your opportunities it’s still often not enough.

This feeling was amplified by his previous status within the academy. “The end of your scholar when you get your contract you end on a high, thinking ‘oh I’m unbeatable.’ Then you train with the first team and you think I have to improve a lot more.”

Oliver continued to find this part challenging.

It’s a bit demoralizing at times. I know on a Friday I’m going to come and I’m going to be put in any position just to replicate the other team. Positions I never play in. It gets a bit frustrating because you want to play where you play and impress. You can’t, you just have to do what they want you to. It’s a bit annoying at times. The word I think is demoralizing. You lose a lot of confidence in yourself.

For Thomas, the introduction to the first team was more positive. “In my first week I think I was quite nervous. Definitely quite nervous. It only takes a week to settle in really. Now I’m ready to start my third week and I feel a lot more comfortable.” The excitement of joining the first team was a boost for Thomas, “being with older pros definitely brings more drive within myself. I need to be at their level and their standard at all times or else they will tell you about it,” adding, “It definitely brings more out of yourself, more life, more buzz and also you want to impress them.” This was important as his transition continued.

If you are at their standard, then you are going to improve. I think just having that drive to be at their standard, I have become fitter and sharper training with men. The tempo is quicker, and you have to be stronger and when people are pressing you, you have to pop it around quicker. I think I have gained sharpness.

He added later, “to be fair I think everyone is a bit worried stepping up but honestly, I feel, that it’s not that much of a challenge. I think I cope with it easy enough.”

##### Social dynamics

In the academy phase Oliver predicted social challenges, which he did indeed experience at the start of this phase, “You don’t really know how to talk to them. I guess that’s where in training you want to earn their respect by training well and if you don’t train well you don’t feel like you can speak to them.” Oliver explored several avenues to integrate including during meals.

I try to sit on other tables with the older lads even if I don’t say anything or know what they are talking about at least I’m there. They know I’m there so maybe sometimes they feel they have to talk to me.

New signings were also a way to have an “in” with the more senior group, “because we have brought in a lot of new lads they know people less than you do so I guess that has been an easy way in, speaking with the new lads.” It was interesting that later on in the study, Oliver seemed less interested in what the experienced pros thought of him.

You think, oh they don’t like me, or you have a bad day and they think you are rubbish. At the end of the day if I make a career out of football and they still think I’m rubbish, I still made a career out of football.

The fact that Charlie was not on loan the previous year helped him be more familiar with the first team environment; “I think the people that I know I’ve talked to a lot. The people I don’t know, that I haven’t really spoken to before, I feel that I’ve gotten to know them a lot more.” His attitude toward adjusting and fitting into the group was shaped by a previous transition.

I’m always genuine, so if anyone doesn’t like what they see of me I feel like I’m not going to change just to please them. I had that many times in the U18’s moving up from the U16’s, things like that. You get players that you just don’t get on with, which is fine because you aren’t going to get on with everyone. I feel it’s important that you stick to who you are, what your morals are, and what you think is right.

Charlie believed that this attitude and willingness to mix in with the group was important to his transition, “I think I have made the transition quite smooth, because I have bonded with players and staff.”

#### Opportunity

##### Loan opportunities

During preseason all three learned they would go on loan for the first half of the season. As this was Charlie’s first loan move, his initial impressions were similar to those of Thomas and Oliver from the previous season. This included acknowledgment of the increased physical demands, “It was more physical, quicker, and sharper”; the style change, “it’s a lot different to what it used to be like in the academy where it was more pass football, pass, pass, pass”; and the competitiveness, “I thought U18’s football was really important at the time but when you are out of it, you look at (the EFL), (U18’s) is not as important as you think it is.”

While Charlie was learning lessons the other two had learned 6 months earlier, Thomas believed that his experiences from his previous loan would be helpful, “I learned a lot about myself and how I can handle things, how I can handle men’s football. I have taken all of that experience.” Further, being on loan a second time shifted priorities from simply learning men’s football to showing the ability to perform. Oliver had a simple goal in mind. “I think if I can cement a good standard of playing at my loan club that will look good for the start of the season to (the manager) and show that I’m fit.” This priority on performance came with a challenge, “now it’s a bit more pressurized. I feel I have to find form and get good stats in games.” Similarly, Thomas targeted being more selfish and achieving performance goals to “get my name out there.” He saw this as key to gaining opportunity at his home club adding, “that’s what is going to get me into the first team.” Oliver had a similar impression, “if I don’t do well when I’m on loan they will probably think, if he can’t play against that standard then how is he going to play against the higher standard?” This perceived pressure remained with Oliver throughout this phase, with him reiterating later in the study, “I definitely feel a lot more pressure.”

The opportunity on loan, again, began to separate the experiences and illuminate potential unintended consequences. Oliver was playing a high number of loan games which he believed helped accelerate his development.

I felt from my loan I had gotten a lot of experience to bring into that (U23’s) game. Just from playing on loan, it’s clearer in my mind what I want to do. You are starting to catch up in terms of football now, with how the first team play at that level of tempo.

While this improvement came as a result of the playing time, Oliver exhibited a high level of self-awareness, adding, “I am finding good techniques to get me up for a game and get me in the right zone to be able to play. I think that is getting better as well, managing myself.”

Meanwhile for Thomas, although the loan period started off positively, “I’ve stamped my authority and showed what I’m about. Hopefully, I can continue and do the best I can do for the team,” he soon became frustrated as playing opportunities became limited.

I thought this is going to be my year. I have worked well in offseason, I had done well in preseason, but they (home club) weren’t really interested. That was a bit of a low. Then I went (on loan) which was a bit exciting, it was a new club to go to. Done well there in preseason, but then didn’t start the first game of the season so that was another thing where I was asking myself why? What have I done?

This feeling was something that he struggled with.

It’s very lonely. Football can be, when you are out of favor, football is a lonely place and I do feel lonely at times. It does get me down. Feeling down about being out on loan because I’m trying my best and not getting the best or what I feel deserve.

He continued, “it’s hard. I don’t know, it’s really horrible. Football is lonely, I don’t know, the experience is hard.”

##### Training experiences

For Charlie, the loan period began to impact how things were going at his home club. “You are out on loan and they think you have another four months out on loan, so we don’t have to worry about him for another four months.” This manifested in training sessions.

You can have a good session and do really well, you are really proud of yourself, and they won’t notice it because they might be doing work with the starting XI for Saturday. They don’t notice how well you are doing. I think you just have to keep going really. People will notice eventually, but it’s obviously hard and a bit degrading really sometimes when you are trying your hardest and putting in a lot of effort, doing well, and it’s not getting noticed.

This frustration was echoed by Thomas, “they aren’t too bothered or focusing on what I’m doing.”

The difficulty is that you are a first team player, but the first team is not really bothered about you. That’s the hardest thing to take. I know that I’m a good player and that I’m good enough, but not being able to get a chance to show what I’m about is the most difficult thing.

Charlie had similar views, “it is hard to maintain confidence because you are in and around the first team, but you aren’t as well.” During this time, he stressed the importance of his loan, “I think being away at the loan club and they feel you are doing well, then that gives you confidence.” At the end of the study he did reflect that perhaps this trade off was worth it, “I feel like I’m readier to play men’s football. I’m readier to compete and win the ball back than when I was an U18. I think that’s the main reason they sent me out on loan.”

Finally, Thomas discussed what he felt was the most important thing he had learned so far.

I’ve learned when you get that opportunity, the higher you go the less opportunities you might get. The less leeway you might get, you might get 45 min then get taken off because that’s not what the manager wanted. It’s cutthroat, but as I get older and as I get more experience, I will be able to know what I have to do to take those opportunities.

For Thomas the day to day challenges seemed to lessen toward the end of the study, nevertheless the major obstacle of lack of opportunity remained.

You can handle the training, you can handle the day to day life, but the challenges that come with trying to get an opportunity or trying to get that break in men’s football is really tough. That’s what I think is the toughest thing for a young kid to get that break.

#### Coping

##### External: support networks

With opportunity limited and frustration mounting throughout the first team phase, access to support was a significant barrier. Oliver outlined a major obstacle to support, “to actually gain attention so that people want to help you, you have to cope with being on your own.” Thomas also felt that more support would help, “I think more support should be available for the young pro’s because it’s so tough for us to get that opportunity. Then, when we get that opportunity, we have to show it or else we will be bombed out.” Charlie had a differing opinion, perhaps created by his staying at the home club previously.

I think it’s important that you know that there are people in the club that would help you. (U23’s manager) would always help you and give you advice. We have teammates, we have family at home, and your loan club. I think it’s important that players know that they aren’t alone and have someone to speak to.

Further Charlie added, “I think some people try to bottle it up.” Something he felt needed to be overcome, “I think some people just struggle to open up to coaches and members of staff, I think that’s the best thing for you really.” In Oliver’s opinion this came with a potential barrier, “It would be nice to have someone in the club that you could speak to, but it’s hard because if you are getting coached by them, you can’t show them your weaknesses.” Whilst Thomas found it difficult for another reason, “you think, I probably do need someone else to speak to about my worries and things like that. You don’t want to be a burden on anyone or think that you are wasting their time or anything.” When Oliver discussed this further, he said, “I don’t think there is really anyone in the club that you can talk to. Obviously, people that I know, but not really people in the club.”

This is something both Thomas and Oliver desired, with Oliver adding, “It would be good because you could express whatever you want to them and they can help you get through it.” While Thomas suggested, “maybe it’s more about them approaching you because you are worried or embarrassed about it.” Further he wanted, “more chats, one on one chats, asking us if we are alright and asking us if we have any worries. That would be good.” This was a large change from the academy where Thomas said, “there are so many people upstairs that you can speak to” and that the 18’s manager, “was always having meetings with you. He was always worrying for you and caring for you because he wants the best.”

Toward the later stages of the study, Thomas felt that coach support did improve.

Recently I have been speaking to our U23’s manager more. Which has been good. He has been telling me what I need to do and what he thinks about me right now, and what the first team manager thinks about me right now. That was a little bit of a confidence booster.

Further, Thomas found one area of social support useful, his peers in the U23’s, “We speak in a group. That’s not really solving anything, that’s just getting your point out there with the 23’s in our changing room. They talk about their experiences and I talk about mine.” He expanded about the importance of this group.

There are a lot of us that are in the same boat. We can give each other encouragement and talk to each other about what we can do to get an opportunity, get better as a player, or stand out on loan.

While this peer group was helpful, Thomas wished for more support from senior players, “you can learn so much from the first team players as well, but it’s getting time to speak to them. Asking them questions and asking them what they did as a youngster. I think that would help.” However, he appreciated that perhaps he would need to take the lead; “I think it’s our job if we really want to go speak to someone from the first team, we have to do it ourselves. I would say I probably haven’t done that as much.”

Oliver shared this difficulty.

Sometimes you feel like they are going to be like, go away. Why are you being so busy? I have to just get my nose in there sometimes. Often, you think that people are going to think, oh look at him being a busy body.

The value of senior player support was expressed by Charlie. “I think it’s important that those players teach you what it’s like to be in the first team dressing room. I think that just listening to them and picking up things that they say, and their ideas is really important.” He continued with a similar idea from the academy phase, “when you get a chance, talk to someone that’s in your position about things they do well and things that they need to improve on. I think that will help you improve your game.” The U23 cup competition was again a helpful resource, “just listening and playing with them who have more experience it’s important to being in and around the first team. You are getting more used to the environment on a match day.” While all three players felt speaking with senior players was important, Charlie was the lone participant to report doing this regularly.

##### Internal: psychology/mindset

As the first team phase continued, important psychological coping strategies were discussed. For Charlie this meant, “just keeping the mindset that if you give 100%, you can’t do any more, that’s a really important mindset that I have thought about. I think other players need to think about that more often.” This mindset remained unchanged throughout this phase, with him adding late in the study, “It’s important that I know that I just need to keep doing my best, keep trying, keep trying to perform to the best that I can. That’s all I can do really.”

Oliver reflected late on in the study that all the challenges he was facing in the transition were a part of his development as a person and player.

I think I have learned to be resilient and I have learned for myself that I’m more resilient. I think I am mentally stronger than I thought I was. Through this, as I’ve said before, in terms of how much you do and how much you train, you are looked after a lot less. You are at the bottom of the pile, so you have to be strong in yourself and look after yourself a lot more.

Further to this he added in the final interview.

Some of those experiences have been good but some of them haven’t been great. I’ve either had to decide for myself, am I going to let this get me down? Or am I going to rise above it and move on to the next?

Despite the frustrations and challenges, Thomas preached one key message throughout this phase.

I think being a young pro you have to wait your time and be patient. That’s hard. Especially, when you want to do your best and everyone is pushing you to try to get that opportunity, but it doesn’t come. You have to be so patient with it. That’s a tough thing to handle. You just have to be patient and bide your time.

He continued, “What’s the point in getting down? You go again, you play well, you train well throughout the week and play for your loan club. See what you can do there.” Similar to the academy phase, Thomas mentioned his resilience, “I have been through ups and downs. I know how to deal with it now.” During this period, he outlined his plan to keep moving forward.

You just have to improve yourself at every little thing, it’s not big steps, it’s just little steps, that’s what I see. Little steps in the gym, little steps on the pitch, little steps off the pitch. That’s just my focus really, to improve myself as a person.

In the latter weeks he seemed to have a more relaxed perspective that allowed him to cope with the challenges he was facing. “I still have time on my contract and I still have people backing me to do well. I am happy with that.” In the final interview he reflected on his time in the academy compared to the challenges he was currently facing.

In the academy you don’t go through any of that, it’s all very comfortable. It’s easy and you aren’t worried about anything, everything is simple. You play a game on a Saturday and that’s it, it’s not like life and death.

## Discussion

The purpose of the current study was to explore the evolving perspectives of young players experiences going through the junior to senior transition in professional football. The innovative video diary approach enhanced the depth of our understanding about the academy to first team transition, specifically providing unique contributions to the literature in the areas of contract decisions; adaptation to senior competition; barriers to transition without early success; and social aspects of the transition. Moreover, the weekly approach to data collection and analysis allowed a clear progression of the experience to be documented.

The transition was initially marked by the pressure to earn the professional contract, a key milestone within the junior-to-senior transition and a necessity for the transition to continue (Drew et al., 2019). The early contract decisions allowed players to begin preparing for life in the first team and focus on their adaptation to men’s football. The first team phase began with the excitement of preseason, including opportunity in first team friendlies, however loan moves quickly shifted the dynamic of this phase. Throughout this phase, players were required to adjust to training demands and a new social dynamic while learning new avenues of formal and informal support from the organization. Being on loan combined with limited opportunity to impress in training at their home club created frustration and led to difficulties in establishing an identity within the first team. Each player relied heavily on personal support networks and their own mindset to cope during this period, as there was limited formal support provided (see Table 3 for themes). Over time these challenges were accepted as part of the process, and players focused on continuing to work toward their goals.

Adapting to senior competition after ten years of youth football was identified early in the academy phase as a priority for all three participants (Morris et al., 2016a). Yet, apart from handling increased mental and physical demands (Morris et al., 2016b), there has been limited enquiry into what players specifically need to adapt *to*. As found in previous research, all three participants initially perceived the physical demands of senior competition to be a challenge (e.g., Morris et al., 2016a; Stambulova et al., 2017). However, this was superseded by difficulties in adapting to a different style of play and the subsequent decision-making demands while being on loan. Time spent on loan and training with the first team helped provide learning opportunities for senior football decisions. Decision making was also crucial, in the short term, to get out of physical battles that would be lost as a younger player.

The focus on the specifics of the adaptation pathways – being on loan and playing with the U23s – was another novel element of the current study. While these pathways are common practice in European football clubs, they have received limited focus in the available literature (see Røynesdal et al., 2018). Participants reported that both provided valuable learning experiences that were not available during their time in the academy. The loan system was the primary method of adapting to the physicality, decision making, and style of play demands of senior football, as well as the distinct focus on winning matches. Participants felt that the focus on winning meant that it was no longer acceptable to take unnecessary risks or play in a way that was previously commended in the academy setting (e.g., Røynesdal et al., 2018). There was pressure to accumulate good match statistics at their loan clubs to show they were impacting games at the senior level and so earn the trust of their club’s senior coaches, something that has been reported previously by ice hockey players (Bruner et al., 2008). While loan moves seem to provide an excellent learning opportunity, they need careful managing if playing time is limited, as was the case for Thomas (see also Røynesdal et al., 2018).

The second pathway – playing for and training at the club with the U23’s – also provided additional, context specific learning opportunities, as they replicated first team style and tactics. It also provided the opportunity to impress first team staff (who coached the U23s) and more experienced first team players (who made up the majority of the U23 match squad) directly. This was not always possible in training with the first team squad, as the focus was on preparing the match day squad for upcoming games. The experiences of being in the changing room with first team players, in first team stadiums, were reported to be valuable to their adaption and motivation.

The next major finding in the current study was the challenges caused because of the lack of opportunity in the first team. This lack of opportunity has been previously reported as a major barrier to successful transitions (Drew et al., 2019), though, the current study provides the first description of how players experience, and learn to cope, with this barrier over the first few months of their transition. Several specific examples were discussed including: motivation and confidence issues; lack of identity with the first team squad; and lack of organizational support. The difficulties with motivation and confidence were discussed throughout the first team phase. Despite not having an opportunity to impress within the first team environment, Charlie and Oliver discussed their loans as a way of maintaining confidence with regular playing time (cf. Bruner et al., 2008; Drew et al., 2019). Without this outlet of playing time on loan, Thomas described feelings of frustration and loneliness.

The use of loan moves as an outlet appears to be a critical organizational strategy for facilitating transitions, especially when it is known there will be a lack of opportunity in the home club (e.g., Pehrson et al., 2017; Drew et al., 2019). Drew et al. (2019) also suggest the importance of organization culture in transitions, as the perception of opportunity can have a large impact on motivation. It is important to note that the club in question has a reputation for providing youth players with opportunity in the first team, though rarely in the first year. At the end of the study the participants were no closer to having such a role (e.g., Morris et al., 2015; Pehrson et al., 2017; Stambulova et al., 2017), and in this example it appears that general adaptation to senior football was prioritized over integration within the first team environment. The impact that this prioritization has on the long-term transition is unclear, but initial negative perceptions were clearly reported, including being demoralized, feeling ignored, and not being supported (see also Morris et al., 2016b).

One key strength of the longitudinal nature of the study was that it was possible to see how participants’ experiences changed over time. Following an initial period of excitement, there was a period of frustration, caused by a lack of involvement in first team training and limited information about what the plan for their development might be. Toward the end of the study period, there was a degree of acceptance; a recognition that patience was important; and reflections on the benefits of the challenging period. Indeed, all three participants reported how the challenges they faced helped make them more “resilient” and tougher, characteristics they knew would be important as they progressed in their careers. Future research should further explore the role of informal peer support (e.g., with colleagues in the U23s), as it appears as though this might be one way in which players can gain perspective through the sharing of similar stories. Toward the end of the study period, all three participants reported that, despite the challenges, they recognized the importance of patience and giving their best, which would lead to opportunity over time. This view could potentially be attributed to the culture of the club that opportunities would become available for young players.

While the informal peer support might have helped the participants gain a sense of camaraderie (they were not alone in feeling frustrated), they did also desire more formal support from the organization. Unfortunately, these formal structures were not readily available (cf. Morris et al., 2015), and Oliver especially felt that he could not approach coaches with his problems as this might be perceived as weakness (Morris et al., 2016b). Charlie was the only participant open to seeking support himself, and comparatively his narrative detailed less challenges, which may be a result of his willingness to seek support (Morris et al., 2016b; Drew et al., 2019). A primary resource for players in the literature has been sport science staff, who provide emotional and informational support about the transition (Morris et al., 2015, 2016a,b). In the current context, the first team had fewer staff members in these roles, especially when compared to the academy, which likely exacerbated the perceived gap in support during the transition.

The final major finding of this study was the enhanced understanding of the social aspects of the academy to first team transition. The competitive environment for places on the match day team was perceived as an obstacle to developing relationships with senior players (Røynesdal et al., 2018), which in turn was related to a circular effect on perceived performance for the participants. There was a belief that being comfortable with senior players was an important precursor to performance on the pitch (Morris et al., 2015; Stambulova et al., 2017), and at the same time performance on the pitch would help in being accepted within the senior team (e.g., Røynesdal et al., 2018). The concept of social competence, believed to be an important psychological predictor of transition success (Drew et al., 2019) may help individuals build relationships despite the competitive environment. The two participants who believed the transition would come with social challenges struggled to build relationships with senior players, while Charlie, who believed he could speak to anyone, was able to.

The important learning from these data for organizations is that simply providing the opportunity for young players to be around senior players is not sufficient for developing relationships (see Larsen et al., 2014). Without an explicit mentor system in place (see Pummell and Lavallee, 2018), there is a greater emphasis on the social skills of each individual (e.g., Drew et al., 2019) and the strength of their own emotional support systems (family, friends, and significant others; e.g., Morris et al., 2016a, b; Pehrson et al., 2017; Drew et al., 2019). One further support network that grew organically in the club was the U23’s group which was particularly important for Thomas. A better understanding of how the dynamic balance of these systems supports successful transitions should be a priority for future research.

## Applied Implications

From an applied perspective, the current study outlines the impact of early contract decisions when comparing the two groups of participants (see Supplementary Files). The early contracts allowed a specific period of preparation with reduced stress and pressure to perform. Organizations should be aware of the timing of contracts when possible, to provide enhanced preparation periods for their players. Further, the adaptation pathways provide important choices for organizations to make in how to best aid adaptation to senior football in general and the specific context of the home club. In this regard, loans benefited the players’ perceptions of adaptation and provided learning opportunities not present in the academy setting (cf. Røynesdal et al., 2018).

Organizations should also seek to understand the experience of players in their context, allowing them to make more informed decisions while balancing intended and unintended consequences. For example, there may be benefits in withholding support if this helps draw out (or reveal) important psychological characteristics such as resilience (Gledhill et al., 2017). However, providing general support as called for by Oliver and Thomas (e.g., a non-coach staff member to talk with) or from targeted support (e.g., a buddy system to help players build relationships with senior players) may increase the likelihood that the transition is successful (Larsen et al., 2014; Pummell and Lavallee, 2018). Avenues of support should be evaluated within organizations and an awareness of players’ views on the ability to seek support. Education in the preparation phase might signpost support pathways for players both internally and externally (Morris et al., 2015).

Specific to the academy system and preparation phase, psychosocial development should be seen as a priority. The development of psychosocial skills can aid in coping with the demands and significant changes that occur during this transition (Stambulova et al., 2020), and in other life domains such as, education, relationships, and future vocation. All three players in this instance discussed specific psychological skills that aided their development and that were used in handling adversity. Social skills should not be overlooked in this process and developing opportunities for interaction between academy and first team players could aid in bridging the gap in transition (Larsen et al., 2014).

## Strengths, Limitations, and Future Directions

This innovative approach to longitudinal research creates opportunities for future researchers to employ similar methods. The video diaries helped drive the collection of a large amount of data with relevantly low amount of time and effort when compared with semi structured interviews. They also allowed flexibility for the participant to complete in their own time what they were most comfortable to share. Weekly data collection allowed enough time to pass for participants to reflect while also providing data in real time, adding nuance to the collected data. As key events were happening during the data collection, participants were able to share their experience “in the moment” providing accounts of their beliefs and the changes over time. Finally, participants discussed with the lead researcher that the video diaries helped provide them with another outlet, creating an additional support source.

Several limitations were present in the current study, which means that the results, while novel, should be interpreted with caution. First, the reduced pre-contract period for three of the participants created a strong disconnect between them and the other three participants during the academy phase of the project. While the timing of contract awards cannot be predicted, this was not common practice in the club and drastically changed the experience that was anticipated by the research group. While dropout was expected, the fact that none of the three participants who received late decisions were successful, meant that we could not compare differences in preparedness for the first team phase within the group. Finally, the organizational context and the specific experiences of this group are critical factors underpinning the transition, and therefore these will be different in each organization. Future research should look to understand the context of their specific organization and examine similarities with, and differences to the findings of the current study.

A methodological aim of this study was to use a novel combination of data collection methods to uncover the experience of those transitioning. Currently, our understanding is limited by the reliance on single time point interviews or focus groups (26 of 27 studies in a recent review by Drew et al., 2019). While semi-structured interviews are staples of qualitative inquiry, especially in sport (Smith, 2010), our experience found that video diaries were easy to implement for both researcher and participant. We encourage future researchers to investigate the use of video diaries, audio diaries, or journals, as methods to examine the *process* of the transition.

This longitudinal exploration illuminated novel details of the transition experience – especially with regards the unfolding and changing nature of the challenges and successes of players as they live this experience yet leaves much still unexplored. Future research should look to explore areas beyond the scope of the current study including the challenges that exist as the transition further develops and players attempt to adjust to the first team squad. Different issues, including those related to social dynamics, are likely to become pertinent as players are brought back from loan and are fighting for their opportunity in their home club. Collecting data on individual transitions across a greater time span comes with challenges of cost and feasibility but could greatly enhance our knowledge of the unfolding ups and downs inherent in the transition.

## Conclusion

The current study explored the evolving experiences of young players over 40 weeks as they navigated the junior to senior transition in professional football. The use of weekly video diaries allowed us to better understand the individual highs and lows encountered as they adapted to life as professional footballers. Of particular interest was the head start in preparation due to early contract decisions, a period that each individual used differently to further their readiness for the full time move to the first team. Upon that move, differences were reported in how the players adapted to the frustrations inherent in receiving limited opportunity to establish themselves on and off the pitch within the first team. While there was no resolution to the source of frustration by the end of the study, the players had developed coping strategies that enabled them to perceive these frustrations as being transient, if they continued to work hard and develop their game. While we have no way of knowing if these players will be successful, or which specific combination of factors would predict their ongoing success we have provided enhanced detail to the specific demands during this transition and how individuals seek to cope with those challenges. The current study provides a launch pad for future studies to adopt similar longitudinal methods that further explore the unfolding experiences of players as they make their way as professional footballers in a way that is free from the bias of retrospective recall.

## Data Availability Statement

The datasets generated for this study are available on request to the corresponding author.

## Ethics Statement

The studies involving human participants were reviewed and approved by the University of Exeter. Written informed consent to participate in this study was provided by the participants’ legal guardian/next of kin. Written informed consent was obtained from the minor(s)’ legal guardian/next of kin for the publication of any potentially identifiable images or data included in this article.

## Author Contributions

SS, MW, and MJ contributed to the study design. SS conducted the data collection. All authors contributed to the preparation of the manuscript.

## Conflict of Interest

The authors declare that the research was conducted in the absence of any commercial or financial relationships that could be construed as a potential conflict of interest.

## Footnotes

1 The experience of the non-contracted players – including their themes and quotations – which were related primarily to the stress and pressure of waiting for the contract decision (see also section “Pressure”), can be found in a Supplementary File.

## References

[B1] Bradshaw C. Atkinson S. Doody O. (2017). Employing a qualitative description approach in health care research. *Glob. Qual. Nurs. Res.* 4 1–8. 10.1177/2333393617742282 29204457 PMC5703087

[B2] Braun V. Clarke V. (2006). Using thematic analysis in psychology. *Qual. Res. Psychol.* 3 77–101. 10.1191/1478088706qp063oa 32100154

[B3] Bruner M. W. Munroe-Chandler K. J. Spink K. S. (2008). Entry into elite sport: a preliminary investigation into the transition experiences of rookie athletes. *J. Appl. Sport Psychol.* 20 236–252. 10.1080/10413200701867745

[B4] Creswell J. W. Poth C. N. (2013). *Qualitative Inquiry and Research Design: Choosing Among Five Approaches*, 3rd Edn Thousand Oaks, CA: Sage Publications.

[B5] Denzin N. K. Lincoln Y. S. (2011). *The SAGE Handbook of Qualitative Research.* Thousand Oaks, CA: Sage.

[B6] Drew K. Morris R. Tod D. Eubank M. (2019). A meta-study of qualitative research on the junior-to-senior transition in sport. *Psychol. Sport Exerc.* 45:101556 10.1016/j.psychsport.2019.101556

[B7] Ducker J. (2017). *Pep Guardiola: Manchester City’s youngstes will not play much next season. The Telegraph.* Available online at: https://www.telegraph.co.uk/football/2017/05/12/pep-guardioloa-manager-citys-youngsters-will-not-play-much (accessed May 12).

[B8] Gledhill A. Harwood C. Forsdyke D. (2017). Psychosocial factors associated with talent development in football: a systematic review. *Psychol. Sport Exerc.* 31 93–112. 10.1016/j.psychsport.2017.04.002

[B9] Grossoehme D. Lipstein E. (2016). Analyzing longitudinal qualitative data: the application of trajectory and recurrent cross-sectional approaches. *BMC Research Notes* 9:136. 10.1186/s13104-016-1954-1 26936266 PMC4776420

[B10] Haugaasen M. Jordet G. (2012). Developing football expertise: a football-specific research review. *Int. Rev. Sport Exerc. Psychol.* 5 177–201. 10.1090/1750984X.2012.677951 30656504

[B11] Larsen C. H. Alfermann D. Henriksen K. Christensen M. K. (2014). Preparing footballers for the next step: an intervention program from an ecological perspective. *The Sport Psychologist* 28 91–102. 10.1123/pes.2013-0015

[B12] Lincoln Y. S. Guba E. G. (1985). *Naturalistic Inquiry.* Thousand Oaks, CA: Sage Publications.

[B13] Mayan M. J. (2009). *Essentials of Qualitative Inquiry.* Walnut Creek, CA: Left Coast Press.

[B14] Morris R. Tod D. Eubank M. (2016a). From youth team to first team: an investigation into the transition experiences of young professional athletes in soccer. *Int. J. Sport Exerc. Psychol.* 15 1–17. 10.1080/1612197X.2016.1152992

[B15] Morris R. Tod D. Oliver E. (2016b). An investigation into Stakeholders’ perceptions of the youth-to-senior transition in professional soccer in the United Kingdom. *J. Appl. Sport Psychol.* 28 375–391. 10.1080/10413200.2016.1162222

[B16] Morris R. Tod D. Oliver E. (2015). An analysis of organizational structure and transition outcomes in the youth-to-senior professional soccer transition. *J. Appl. Sport Psychol.* 27 216–234. 10.1080/10413200.2014.980015

[B17] Morse J. (2020). The changing face of qualitative inquiry. *Int. J. Qual. Methods* 19 1–7. 10.1177/1609406920909938

[B18] Neergaard M. A. Olesen F. Andersen R. S. Sondergaard J. (2009). Qualitative description - the poor cousin of health researcher? *BMC Med. Res. Methodol.* 9:52. 10.1186/1471-2288-9-52 19607668 PMC2717117

[B19] Pehrson S. Stambulova N. B. Olsson K. (2017). Revisiting the empirical model ‘Phases in the junior-to-senior transition of Swedish ice hockey players’: external validation through focus groups and interviews. *Int. J. Sports Sci. Coach.* 12 747–761. 10.1177/1747954117738897

[B20] Pummell E. Lavallee D. (2018). Preparing UK tennis academy players for the junior-to-senior transition: development, implementation, and evaluation of an intervention program. *Psychol. Sport Exerc.* 40 156–164. 10.1016/j.psychsport.2018.07.007

[B21] Rich M. Lamola S. Gordon J. Chalfen R. (2000). Video intervention/prevention assessment: a patient-centered methodology for understanding the adolescent illness experience. *J. Adolesc. Health* 27 155–165. 10.1016/S1054-139x(00)00114-2 10960213

[B22] Røynesdal Ø Toering T. Gustafsson H. (2018). Understanding players’ transition from youth to senior professional football environments: a coach perspective. *Int. J. Sports Sci. Coach.* 13 26–37. 10.1177/1747954117746497

[B23] Sandelowski M. (2000). Whatever happened to qualitative description? *Res. Nurs. Health* 23 334–340. 10.1002/1098-240X(200008)23:4<334::AID-NUR9>3.0.CO;2-G 10940958

[B24] Sandelowski M. (2010). What’s in a name? Qualitative description revisted. *Res. Nurs. Health* 33 77–84. 10.1002/nur.20362 20014004

[B25] Schlossberg N. K. (1981). A model for analyzing human adaptation to transition. *Couns. Psychol.* 9 2–18. 10.1177/001100008100900202

[B26] Smith B. (2010). Narrative inquiry: ongoing conversations and questions for sport and exercise psychology research. *Int. Rev. Sport Exerc. Psychol.* 3 87–107. 10.1080/17509840903390937

[B27] Smith B. (2018). Generalizability in qualitative research: misunderstandings, opportunities and recommendations for the sport and exercise sciences. *Qual. Res Sport Exerc. Health* 10 137–149. 10.1080/2159676X.2017.1393221

[B28] Smith B. Caddick N. (2012). Qualitative methods in sport: a concise overview for guiding social scientific resaerch. *Asia Pacific J. Sport Soc. Sci.* 1 60–73. 10.1080/21640599.2012.701373

[B29] Sparkes A. C. Smith B. (2009). Judging the quality of qualitative inquiry: criteriology and relativism in action. *Psychol. Sport Exerc.* 10 491–497. 10.1016/j.psychsport.2009.02.006

[B30] Stambulova N. (2003). “Symtpoms of a crisis transition: a grounded theory study,” in *Svensk Idrottspsykologisk Förening*, ed. Hassmen N. (Hyderabad: University Press), 97–109.

[B31] Stambulova N. Alfermann D. Statler T. Côté J. (2009). ISSP position stand: career development and transitions of athletes. *Int. J. Sport Exerc. Psychol.* 7 395–412. 10.1080/1612197X.2009.9671916

[B32] Stambulova N. Pehrson S. Olsson K. (2017). Phases in the junior-to-senior transition of Swedish ice hockey players: from a conceptual framework to an empirical model. *Int. J. Sports Sci. Coach.* 12 231–244. 10.1177/1747954117694928

[B33] Stambulova N. Ryba T. V. Henriksen K. (2020). Career development and transitions of athletes: the international society of sport psychology positions stand revisisted. *Int. J. Sport Exerc. Psychol.* 2020:1737836 10.1080/1612197X.2020.1737836

[B34] Sullivan-Bolyai S. Bova C. Harper D. (2005). Developing and refining interventions in persons with health disparities: the use of a qualitative description. *Nurs. Outlook* 53 127–133. 10.1016/j.outlook.2005.03.005 15988449

[B35] Wylleman P. Lavallee D. (2004). “A developmental perspective on transitions faced by athletes,” in *Developmental sport and exercise psychology: A lifespan perspective*, ed. Weiss M. R. (Morgantown, WV: Fitness Infomration Technology), 502–523.

