# Supplementary material for: Player Experience During the Junior to Senior Transition in Professional Football: A Longitudinal Case Study
Source: Front Psychol. 2020 Jul 9;11:1672. doi: 10.3389/fpsyg.2020.01672 (PMC7363945; doi:10.3389/fpsyg.2020.01672)
Supplement: Supplementary file 1 [file Data_Sheet_1.pdf]

Supplementary Material

1 Released players

Three players were not offered professional contracts following their two-year apprenticeship. John was informed of this decision during week 8 of the study while David and George were informed at the end of season reviews during week 13. Over the course of the study these three players reported dealing with a pressure to perform on the pitch in order to earn a professional contract (see table 3). All three players reported their goal was to play professional football and that this was something that they had been working towards since they joined the youth academy. While they all reported this pressure as a part of their experience, they had individual differences in how it was perceived, as well as how they attempted to cope with the pressure to earn a contract.

1.2.1 John

From the first week of the study John described the pressure of the contract decision, “it’s difficult because we are coming to decisions soon. I need to make sure I am playing well as often as I can.” While the pressure was present in week one so to was his focus on his future as a whole, “I want to make sure I have all my options open.” For him this meant exploring other options such as university, because, “I don’t want to be in a position where I don’t earn a contract and have nothing to fall back on.” Throughout the study the balance of these two areas were at the center of John’s narrative.

The pending decision resulted in him counting down sessions, saying, “if you have a bad session you think that’s one session I’ve wasted.” The pressure combined with the limited time remaining led to difficulty when interpreting mistakes, “you have that thought of, if I make a mistake, they are going to use it against me, that helps towards their decision. It’s difficult.” Following his release, he reflected on the pressure.

> You are having that fear of are you going to make it? Am I going to get the contract? It wasn't like I lost enjoyment, but it was frustrating. If you have a bad session, that's one less session that you can prove yourself. The enjoyment was there, well, in terms of the fear it does put that enjoyment down a bit.

John believed that coping with pressure was a key factor in the transition and earning a contract, however, it was not something he learned until late in his time in the academy. "I think it's how you deal with the pressure. How you either use it as an advantage or not." One way John attempted to cope with this was by exploring his career options, which had a positive influence both on and off the pitch. "Psychologically, I think it's allowed me to play really good football knowing that if it doesn't happen, I have somewhere else to go", and later, "it reassured me that I can go out and play with freedom." Further, he described how he might feel without the security of knowing he had other options, "if this was all I was going for then I would probably be very uptight, very stressed, very worried about making mistakes, and having a bad training session."

1.2.2 George

In week 3, George stated he was behind in earning a contract. "I have a lot to do to get a professional contract." That same week George admitted, "If I'm being honest, I don't think I am going to get a professional contract" citing injury and performance. Also, in week 3, George was looking into other options including university.

> It's really important. You need something to do. The amount of people that become a professional footballer is so slim compared to the amount of people who try. If you have a plan B, it's there now, you just focus on your football. You don't have to worry about what you would do if you weren't to get a contract.

For George the pressure was a result of the environment in elite football. "It's so intense every day, I lose the enjoyment of it and that's why I make mistakes and don't play as well."

However, in his mind the pressure in the environment was likely to be ever present in elite football.

> You will always be in high pressure environments. If you crumble under the environment, then you aren't going to make it to the very top. If you succeed under the high pressure, show what you can do and what you are best at then you are going to see yourself progressing.

As a result, George believed the pressure led to a choice on how you approach this period. "It is mentality. Do you worry more about making a mistake or not worry if you do make a mistake?" Throughout the study, George linked pressure to enjoyment and his view of mistakes.

> I almost need to forget or not forget, but not think about the pressure of me not getting a pro and just enjoy the last few months. That's when I'm playing the best when I am enjoying football.

Towards the end of the study, he reflected that he learned these coping strategies late in the process, "I reckon I could have developed this sooner" and "it's taken me awhile to realize it, but when I did realize it, I've enjoyed it a lot more."

1.2.3 David

David described how the pressure impacted him early on.

> I try not to stress when things don't go my way. Recently, I've been feeling when I haven't trained well that I get stressed. I think that's one more session down, another reason for them not to give me one.

This counting down of sessions led to the stress growing over the course of the study. "When you don't perform well your stress goes crazy, because there isn't long before decisions are made. You get a bit worried thinking, are they interested in me?" This seemed to continuously

grow, “when I think that’s the last review before (the decision) that makes it even more stressful.”

> Off the pitch the stress levels have gone crazy. I don’t think about it, but I can feel myself being stressed. I think why am I stressed? My first thought is football and then I find myself thinking about it for ages.

According to David, the pressure was part of the environment, “one of the hardest things about what we do, the scholarship, is the pressure you are under. In football in general the pressure is quite immense and hard to deal with at times.”

Coping with the pressure was a major challenge for David. “I haven’t really come across anything that I think, oh yeah, I have to do that because it works.” This lack of coping extended to his willingness to seek support from his family. “I knew that they would be upset for me and be worried. I wanted to stay strong to keep them happy.” He also believed support couldn’t be sought within the club. “You have to be careful telling them how you feel” and stating that, “they might see it in a negative way with how you are feeling if you tell them something, that can be difficult to speak about. Obviously, you want them to have the best opinion of you as you can.” Finally, another coping strategy, seeking a plan B was not an option for David. “I can’t think about it too much because I think I have then accepted failure.”

2 Summary

The pressure to perform was something that all six participants discussed, however due to the lack of contracts awarded to these three participants, further insight is available about the role of pressure in the transition. They all believed performance in training sessions and matches was the primary determinate of earning a professional contract. This, in large part, meant proving their abilities on a daily basis to stand out. The three non-contracted players struggled with the pressure and how they reflected on their own performance in training. David and

John reported high levels of stress as they felt time running out to prove they should earn a contract, while George reported losing enjoyment due to the high-pressure environment.

Several coping strategies were reported including having a plan B and mindset. John and George both developed a plan B, which in their view helped them feel more relaxed and play with more freedom. David chose not to explore future options, believing it was giving up. All three believed dealing with this pressure was a key characteristic in earning a contract. John and George both believed they improved their mindsets to deal with the pressure from the environment, however, in both cases it seemed to be tied to their release or feeling they wouldn’t earn a contract.

In closing, these findings add to our understanding of the pressure encountered prior to earning a contract. The three participants who earned contracts mentioned pressure as a factor, however each believed the majority of that was relieved by the early contract as they shifted their attention to adapting to men’s football and preparing for the first team environment. The remaining three, without contracts, detailed their feelings and challenges with the pressure of limited time remaining to earn a contract. In their narratives there was limited discussion of preparation and adaptation to senior football as they were primarily focused on proving they should earn a contract. However, as none of these participants received contracts the ramifications of this as it relates to their continuing transition is unknown.

Supplementary Material Table 1. Higher and lower order themes for non-contracted players.

Phase	Higher order themes	Lower order themes
Academy phase – no contract	Pressure	View of mistakes<br>Plan B<br>Coping<br>Limited time
